# Another Conviction for Substitution

**Published:** 1905-02

**Authors:** 


### Another Conviction for Substitution.

THE *Canadian Journal of Medicine and Surgery* in its edition for January, 1905, comments as follows:

Kress & Owen vs. Cruttenden.—On the eighth day of December, Police Magistrate Denison, in the police court, registered a conviction against Thomas Cruttenden, Jr., who keeps two drug stores in Toronto; one at the corner of Howard and Sherbourne streets, and the other at the corner of Gerrard and Sumach streets, for infringement of the trade mark, duly registered in Canada, owned by Kress & Owen Company, 210 Fulton street, New York, "glyco-thymoline." The evidence conclusively showed that the defendant had put up a preparation under the name of "glyco-thymol," in bottles almost identical to those of Kress & Owen

Company, and with labels worded verbatim et literatim to those of the original manufacturers. The magistrate, in registering the conviction, gave the defendant's solicitor, who hinted at an appeal, to understand that, if he entertained that idea, he would not only fine but imprison his client as the law provided. The case was adjourned for a week, at the end of which time Cruttenden, through his solicitor, gave an undertaking that he would stop all manufacture of glyco-thymol and destroy all labels, bottles, and the like, connected with the sale of that preparation.

The firm of Kress & Owen Company are deserving of congratulation over the result of this case. They had every reason for prosecuting Cruttenden, as it was nothing short of dishonest, and entirely contrary to the law, that he should stoop to such practises and try to rob a firm who, by strictly ethical advertising (solely to the profession) and the expenditure of about $175,000 per annum, have secured a large sale of glyco-thymoline, a preparation found valuable in catarrhal conditions of the mucous membrane.

